# Effect of Platelet-Derived Microparticles on the Expression of Adhesion Molecules in Endothelial Cells

**DOI:** 10.3390/ijms26146567

**Published:** 2025-07-08

**Authors:** Elvira Varela-López, Socorro Pina-Canseco, Felipe Massó-Rojas, Claudia Lerma, Ana María Mejía Domínguez, Jesús Oswaldo García Ávila, Juan Carlos Torres-Narváez, Alvaro Vargas-González, Araceli Páez-Arenas

**Affiliations:** 1 Laboratorio de Medicina Traslacional, Unidad de Investigación UNAM-INC, Instituto Nacional de Cardiología Ignacio Chávez, Mexico City 04480, Mexico; varelopz@yahoo.com (E.V.-L.); f_masso@yahoo.com (F.M.-R.); 2 Centro de Investigación, Facultad de Medicina-UNAM-UABJO, Universidad Autónoma “Benito Juárez” de Oaxaca, Oaxaca 68020, Mexico; 3 Departamento de Biología Molecular, Instituto Nacional de Cardiología Ignacio Chávez, Mexico City 04480, Mexico; dr.claudialerma@gmail.com; 4 Banco de Sangre, Instituto Nacional de Cardiología Ignacio Chávez, Mexico City 14080, Mexico; mejana@cardiologia.org.mx; 5 HIV/STI Prevention Department, Fort Bend Country Health Department, Richmond, VA 77469, USA; waldo_basingseto353@icloud.com; 6 Departamento de Farmacología, Instituto Nacional de Cardiología Ignacio Chávez, Mexico City 04480, Mexico; juancarlostn63@hotmail.com; 7 Departamento de Fisiología, Instituto Nacional de Cardiología Ignacio Chávez, Mexico City 04480, Mexico; alvaro.vargas@cardiologia.org.mx

**Keywords:** adhesion molecules, platelets, microparticles, endothelial cells

## Abstract

In healthy conditions and cardiovascular diseases, the most abundant microparticles (MPs) in the bloodstream are those of platelet origin, but the direct effect of these microparticles on endothelial activation is poorly understood. The objective of this paper is to measure endothelial cell activation, as evaluated by the expression of the adhesion molecules E-selectin, VCAM-1, ICAM-1, and PECAM-1 in endothelial cell line HMEC-1 when stimulated with MPs produced by platelets stimulated in vitro with thrombin (TH), adenosine diphosphate (ADP), calcium ionophore (ICa), N-acetylglucosamine (NAcGlc), and without any stimulus. Platelets from healthy individuals induced the formation of MPs with different agonists. The results from the determination of the phenotype of the MPs showed that the expression of GPIIb/IIIa was significant, with median fold changes of TH = 2.2, ADP = 5.2, Ica = 7.0, and NAcGlc = 10.0. However, in HMEC-1 cells, the expression of adhesion molecules stimulated with MPs had a median change slightly higher for E-Sel expression (ranging from 1.4 to 4.2) and ICAM-1 expression (range 2.2 to 3.0), especially VCAM-1 expression (ranging from 15 to 18.8), all of which were significant. For PECAM-1, only stimulation with ICa (1.5) was significant, demonstrating that MPs elicit stimulus-dependent responses in endothelial cells. Platelet-derived MPs may have a potential role in modulating inflammation and other endothelial functions.

## 1. Introduction

Platelets play a fundamental role in hemostasis and thrombosis. In hemostasis, platelets are activated in response to vascular damage to control bleeding [1]. However, their role is vital in cardiovascular diseases, such as myocardial infarction, since platelets form the thrombus, contributing to blood vessel occlusion [2].

Platelets interact with endothelial cells and leukocytes. Platelets are essential in recruiting leukocytes to the injury site in cardiovascular diseases such as myocardial infarction and heart failure. The molecular processes for leukocyte recruitment in postcapillary venules are mediated by inflammatory molecules, such as histamine or tumor necrosis factor-alpha (TNF-α), which activate endothelial cells. This activation results in the expression of adhesion molecules; the first to be expressed are P-selectin and E-selectin, which induce short-lived interactions that induce the rolling process of leukocytes on endothelial cells. The next step is firmer adhesion to the endothelium by the expression of vascular cell adhesion molecule-1 (VCAM-1) and intercellular adhesion molecule-1 (ICAM-1), followed by transmigration. Platelet adhesion molecule (PECAM-1) is present both in the platelet membrane and the endothelial cell membrane and participates in the junctions between endothelial cells and the transmigration of leukocytes [3].

When a vessel wall is damaged, matrix components in the subendothelium, such as collagen and von Willebrand factor, are exposed, and circulating platelets bind to it through glycoproteins in the platelet membrane. This contact initiates platelet activation, with the activation of the fibrinogen receptor αIIbβ3 (GPIIb/IIIa) integrin and the collagen receptor α2β1 [4]. Through intracellular signaling processes, this activation aims to propagate the response to other platelets, thereby stopping bleeding through thrombus formation. However, it has been observed that endothelial denudation is not a prerequisite for platelets to bind to the arterial wall. This has been demonstrated by studies in human umbilical vein endothelial cells (HUVECs), where platelets bind to the cell monolayer, stimulated with interleukin 1 (IL-1), through the ICAM-1 receptor, also known as integrin αvβ3, and GPIb [5].

Platelets, when activated, initiate an intracellular signaling process that goes from the change of platelet shape, expression, and conformation change of glycoproteins and membrane receptors, such as P-selectin and GPIIbIIIa, to the release of a wide range of biomolecules including more than 300 proteins (such as growth factors, chemokines, adhesion ligands) nucleotides and neurotransmitters [6], contained in substructures known as alpha granules and dense granules [7]. Another relevant derivative of platelets are microparticles (MPs), also called “platelet powder” [8]. Platelet-derived microparticles have been observed to exhibit a procoagulant function. Their participation in the pathogenesis of cardiovascular, cerebrovascular, hematological, infectious, autoimmune, cancer, and diabetes diseases, among others, has been demonstrated [9]. Large amounts of circulating platelet-derived MPs have been found in patients with carotid atherosclerosis and myocardial infarction [10], and have also been implicated in the development and progression of atherosclerosis, the underlying cause of many cardiovascular diseases [11].

Microparticles are shed from the plasma membrane of countless cell types by budding; they are heterogeneous sacs capable of encapsulating and transferring multiple types of content, including proteins, RNA transcripts, and miRNAs [10]. Platelet-derived MPs are formed after activation, prolonged storage, and even in resting platelets [12]. The type of stimulus that induces MPs’ release also determines their constituents and content; therefore, MPs generated by cell activation or apoptosis may express different surface markers and inner molecules. Platelet MPs express endothelium-platelet adhesion molecule (PECAM-1, CD31), CD62 (P-selectin), glycoproteins IIb/IIIa (GPIIb/IIIa), P-selectin/CD42a, CD41 (GpIb), and CD63 [13]. P-selectin is widely used as a platelet activation marker [14]. This molecule allows for the interaction of platelets with activated endothelial cells [15].

N-acetylglucosamine (NAcGlc) is an amino sugar categorized as essential for human life [16]. This monosaccharide is part of more complex oligosaccharides found on the surface of erythrocytes and in the extracellular matrix of animal cells, as well as in components of the bacterial and fungal cell walls [17,18]. This sugar has been tested as a promising treatment for various diseases: inflammatory bowel diseases such as Crohn’s disease, ulcerative colitis, and severe lung inflammation caused by human adenovirus type 2; degenerative diseases such as rheumatoid arthritis and osteoarthritis; autoimmune diseases such as multiple sclerosis; and viral respiratory diseases [18]. There is evidence that N-acetylglucosamine polymer fibers can irreversibly activate platelets, inducing the expression of P-selectin, PS, and GPIIb/IIIa, and are used to stop hemorrhage in surgical procedures [19,20].

The relevance of MPs lies not only in their procoagulant and prothrombotic capacity, the latter being mediated by phosphatidylserine in the microparticle membrane, where coagulation enzyme complexes such as the prothrombinase complex are coupled [1], but also in the transmission of regulatory signals [16]. Although it has been reported that platelets increase MP production when stimulated by thrombin–collagen, adenosine diphosphate (ADP), and calcium ionophore, the effect of other agonists such as the carbohydrate N-acetylglucosamine (NAcGlc) is unknown. Furthermore, the effect of these microparticles generated by the stimulation of different agonists in endothelial cells, particularly on the expression of adhesion molecules, has not been evaluated. Although the number of platelet-derived microparticles increases after an acute myocardial infarction [21], the role these microparticles play in endothelial function remains unclear. We propose that different stimuli induce microparticles with different activities. This would suggest that not all microparticles, even those of the same origin, induce the same response, and that the role of microparticles can vary depending on the stimulus that originated them. This could help explain the role played by platelet-derived microparticles in different cardiovascular diseases.

Currently, MPs are of interest as potential biomarkers due to their direct role in the pathophysiology of various diseases and as a potential therapeutic target. The objective of this study was to examine the effect of platelet-derived microparticles activated by different agonists (thrombin, ADP, calcium ionophore N-acetylglucosamine) on the expression of adhesion molecules such as ICAM-1, VCAM-1, PECAM-1, and E-selectin in endothelial cells, and to compare these effects with each other. Among the agonists, we used physiological agonists such as ADP and thrombin, as well as the amino sugar NAcGlc.

## 2. Results

### 2.1. Phenotypes of Microparticles Generated Using Different Agonists

Platelets separated into five groups were stimulated with thrombin, ADP, calcium ionophore, and NAcGlc, or were without stimulation. Microparticles were isolated, and, for their characterization, the number of MPs from each sample was quantified using cytometry using an aliquot of 5 µL. The median value quantified of the MPs without an agonist was 402,062 units, in MPs with thrombin was 391,139 units, in MPs with ADP was 404,702 units, in MPs with ICa was 381,118 units, and in MPs with NAcGlc was 370,908 units. The Kruskal–Wallis test was performed, and no significant differences were found between the concentrations of microparticles obtained with each agonist.

**Figure 1:**
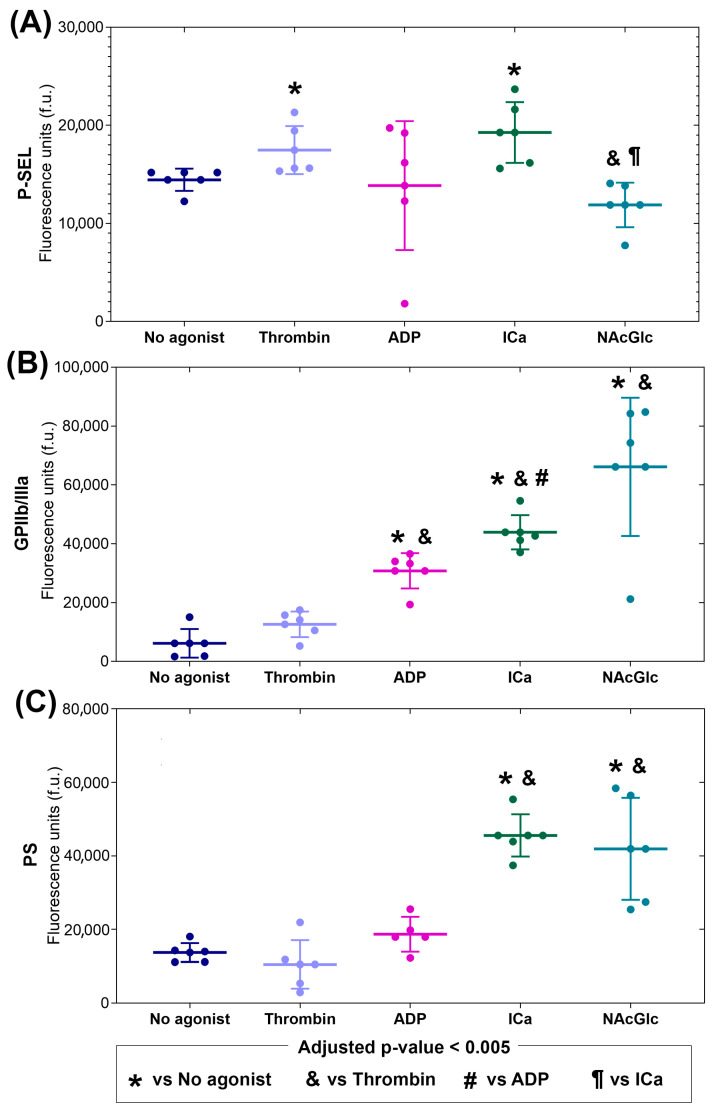
Phenotype of membrane surface molecules (N = 6) expressed on the platelet microparticles: (**A**) P-selectin, (**B**) active GPIIb/IIIa, and (**C**) phosphatidylserine (PS). The microparticles were generated by platelets exposed to different agonists: thrombin, adenosin dyphospate (ADP), calcium ionophore (ICa) and N-acetylglucosamine (NAcGlc). The data is shown as the median with an interquartile range and individual points.

The MP membrane surface expression of P-selectin is characterized in Figure 1A. P-selectin expression on microparticles produced by NAcGlc stimulation was significantly lower than that generated without agonist or ICa stimulation. P-selectin expression did not differ from the other stimuli. P-selectin expression on MPs generated by thrombin and ICa was significantly higher compared to MPs generated without an agonist. The *p*-values from all comparisons are shown in the Supplementary Materials (Table S1).

The MP membrane surface expression of GPIIb/IIIa is shown in Figure 1B. GPIIb/IIIa expression stimulated with ADP, ICa, and NAcGlc was higher than microparticles generated without an agonist or thrombin stimulation. Furthermore, GPIIb/IIIa expression was even higher under ICa stimulation than ADP stimulation. In contrast, GPIIb/IIIa expression in thrombin-stimulated microparticles was not different from microparticles generated with no agonist. The *p*-values from all comparisons are shown in the Supplementary Materials (Table S2).

The MP membrane surface expression of PS is shown in Figure 1C. PS expression in MPs generated with ICa and NAcGlc was significantly higher than in MPs generated without an agonist and thrombin. PS expression in MPs generated with thrombin and ADP did not show significance. The *p*-values from all comparisons are shown in the Supplementary Materials (Table S3).

To provide a clear comparison of the surface marker expression (P-selectin, GPIIb/IIIa, and PS) on the microparticles across the different agonist groups, Table 1 shows the magnitude of response (median fold-change) compared to the expression without an agonist. While thrombin and ADP only increased the expression of P-selectin and GPIIB/IIIa, respectively, the stimulus with Ica increased the expression of all surface molecules. NAcGlc increased the expression of both GPIIB/IIIa and PS, with a stronger effect shown in the expression of GPIIb/IIIa (median-fold change of 11.4).

### 2.2. Expression of Adhesion Molecules on Endothelial Cells in the Presence of MPs

**Figure 2:**
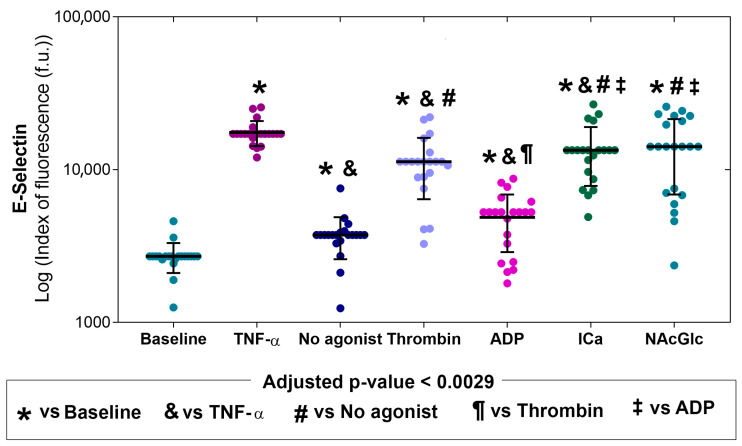
Expression of E-selectin on the membrane of endothelial cells (N = 21) without stimulation (baseline), with stimulation (TNF-α), and after exposure to microparticles derived from platelets activated with different agonists (no agonist, thrombin, ADP, ICa, and NAcGlc). The data is shown as the median with an interquartile range and individual points.

Figure 2 shows the expression of E-selectin on endothelial cells under different study conditions. The first two bars of each graph show the level of adhesion molecule expression in the absence of stimulation (basal) or when stimulated with TNF-α (positive control). The last five bars of each graph show the level of adhesion molecule expression on endothelial cells exposed to platelet-derived microparticles with no agonist or stimulated by thrombin, ADP, ICa, and NAcGlc. Compared with basal (*), E-selectin expression was higher when with no agonist, thrombin, ADP-, ICa-, and NAcGlc-stimulated microparticles. E-selectin expression was significantly higher on platelet microparticles produced with thrombin, ICa, and NAcGlc than on microparticles generated without an agonist (symbol #). Similarly, endothelial cells stimulated with ICa- and NAcGlc-generated microparticles had higher E-selectin expression than those stimulated with ADP-generated microparticles (symbol ‡). E-selectin expression decreased on the endothelium when stimulated with ADP-generated MPs compared to thrombin (¶). *p* values for comparisons between stimuli are shown in the Supplementary Materials (Table S4). Representative flow cytometry histograms of the expression of E-selectin across all stimuli are shown in the Supplementary Materials (Figure S1).

**Figure 3:**
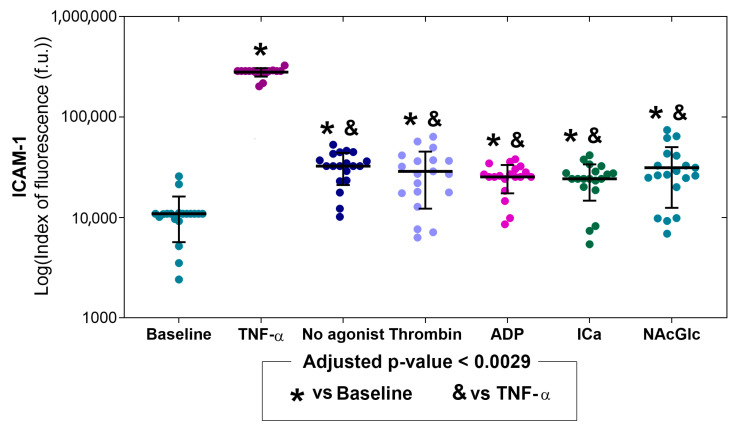
Expression of ICAM-1 on the membrane of endothelial cells (N = 19) without stimulation (baseline), with stimulation (TNF-α), and upon exposure to microparticles derived from platelets activated with different agonists (no agonist, thrombin, ADP, ICa, and NAcGlc). The data is shown as the median with an interquartile range and individual points.

Figure 3 shows ICAM-1 expression in endothelial cells under different study conditions, with basal or TNF-α stimulated expression levels in the first two bars and microparticle-stimulated expression levels in the next five bars. ICAM-1 expressions stimulated with microparticles produced without an agonist, thrombin, ADP, ICa, and NAcGlc were significantly higher compared to basal (indicated with *). In all microparticle exposures, without agonist and with all stimuli, ICAM-1 expression was lower than in the positive control with TNF alpha (symbol &). ICAM-1 expression levels were similar in all microparticle exposures (without agonist, thrombin, ADP, ICa, and NAcGlc). *p* values for comparisons between stimuli are shown in the Supplementary Materials (Table S5). Representative flow cytometry histograms of the expression of ICAM-1 across all stimuli are shown in the Supplementary Materials (Figure S1).

**Figure 4:**
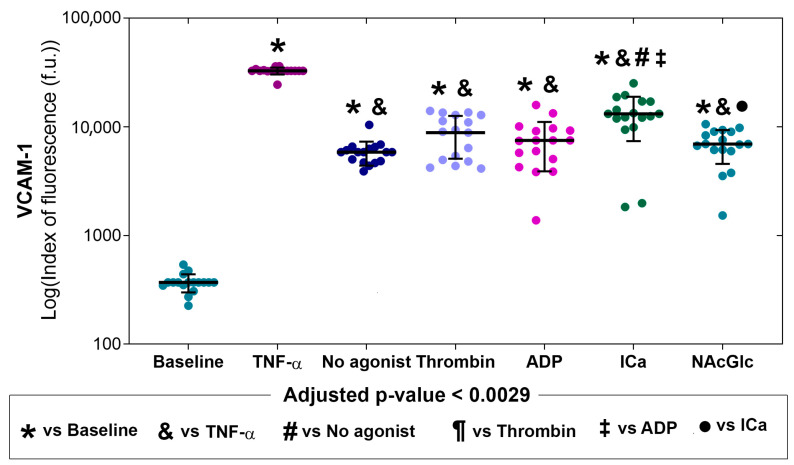
Expression of VCAM-1 on the membrane of endothelial cells (N = 17) without stimulation (baseline), with stimulation (TNF-α), and upon exposure to microparticles derived from platelets activated with different agonists (no agonist, thrombin, ADP, ICa, and NAcGlc). The data is shown as the median with an interquartile range and individual points.

Figure 4 shows that the expression of vascular cell adhesion molecule-1 (VCAM-1) in endothelial cells stimulated with microparticles generated by thrombin, ADP, ICa, NAcGlc, and without an agonist was significantly higher compared to the basal (indicated with *). The microparticles obtained from the ICa produced a greater expression of VCAM-1 in endothelial cells compared to the stimulation of microparticles generated without an agonist (symbol #), by thrombin (symbol ¶), and with ADP (symbol ‡). The stimulation with microparticles with NAcGlc produced a lower expression of VCAM-1 than with microparticles generated by ICa (symbol ●). *p* values for comparisons between stimuli are shown in the Supplementary Materials (Table S6). Representative flow cytometry histograms of the expression of VCAM-1 across all stimuli are shown in the Supplementary Materials (Figure S1).

**Figure 5:**
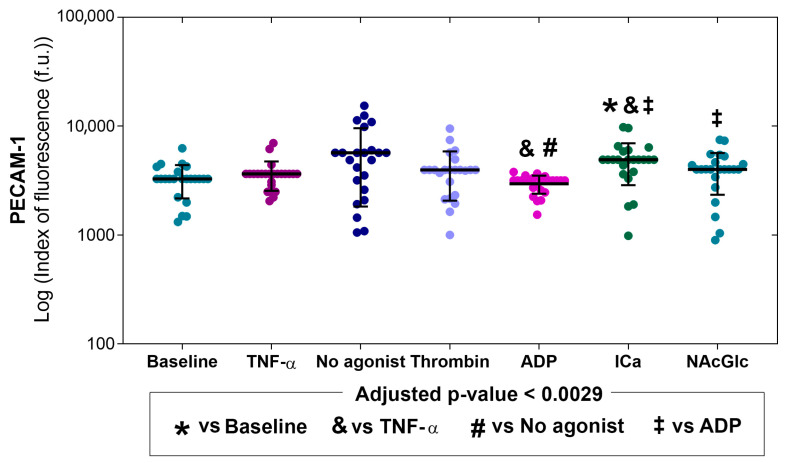
Expression of PECAM-1 on the membrane of endothelial cells (N = 23) without stimulation (baseline), with stimulation (TNF-α), and upon exposure to microparticles derived from platelets activated with different agonists (no agonist, thrombin, ADP, ICa, and NAcGlc). The data is shown as the median with an interquartile range and individual points.

Figure 5 shows that the expression of PECAM-1 in endothelial cells stimulated with microparticles generated with ADP was lower compared to microparticles produced by platelets without an agonist (symbol #). Finally, endothelial cells stimulated with microparticles derived from platelets with ICa and NAcGlc expressed a higher PECAM-1 than cells stimulated with microparticles generated by ADP (symbol ‡). Microparticles generated by ADP significantly decreased PECAM expression compared to TNF-α stimulation. PECAM expression was significantly increased with ICa-generated MPs compared to baseline and TNF-α stimulation. *p* values for comparisons between stimuli are shown in the Supplementary Materials (Table S7). Representative flow cytometry histograms of the expression of PECAM-1 across all stimuli are shown in the Supplementary Materials (Figure S1).

Table 2 summarizes the increase in adhesion molecule expression (E-selectin, ICAM-1, VCAM-1, and PECAM-1) in response to microparticles (MP) derived from stimulation by different agonists. The stimulus by MP derived by ICa increases the expression of all adhesion molecules, even PECAM-1, with a mild response (median fold-change of 1.5). All other stimuli increase the expression of E-selectin, ICAM-1, and VCAM-1, with the strongest effect on VCAM-1 (median fold-changes above 15 in response to all stimuli). The response in the expression of ICAM-1 was homogeneous, with moderate effects (median fold-changes between 2.0 and 3.0). In contrast, E-selectin had a heterogeneous mild response (median fold-changes lower than 2.0) to MPs derived from no agonist and ADP, and a moderate response (median fold-changes between 4.0 and 5.2) to thrombin, ICa, and NAcGlc.

## 3. Discussion

### 3.1. Main Contribution

Our findings reveal that the profile of microparticles generated in response to the stimuli exhibited a distinct expression of P-selectin, GPIIb/IIIa, and PS, suggesting that the composition of microparticles (i.e., the packaging of proteins and other molecules) is agonist-dependent and that different platelet activation pathways generate them. Furthermore, we observed a distinct effect on the expression of adhesion molecules in endothelial cells stimulated by the various groups of microparticles. This response, as revealed by endothelial cell adhesion molecules, suggests that platelet-derived microparticles may regulate endothelial function. Studies using platelet-derived MPs using flow cytometry and confocal microscopy found that they induced an increase in ICAM-1 expression in endothelial cells, in addition to the production of IL-8, IL-1b, and IL-6 [22]. Furthermore, platelet-derived MPs were found to induce angiogenesis in a rat infarction model. In an in vitro model with HUVECs, platelet-derived MPs stimulated endothelial cell proliferation and tube formation [23].

### 3.2. Characterization of Platelet-Derived Microparticles

The conformational change in GPIIb/IIIa in platelets plays a crucial role in platelet adhesion and aggregation. Following activation by ADP or thrombin, GPIIb/IIIa undergoes a shift from a resting to an activated conformation, resulting in fibrinogen binding and platelet aggregation [24]. The results of the present study show that GPIIb/IIIa activation in microparticles was markedly elevated in response to ADP, ICa, and NAcGlc (Figure 2). This indicates that the microparticles generated with the three stimuli exhibited an aggregating function, i.e., the microparticles present activated GPIIb/IIIa.

Platelet activation by the ICa also produces microparticles with activated integrin (GPIIb/IIIa). The increase in activated GPIIb/IIIa with ICa was greater than that with ADP. Stimulation with ICa represents a robust signal that can act on several signaling pathways. Among the signaling events in which it participates are granule secretion, PS translocation to the outer membrane, and microparticle production [25]. This suggests that ICa even activated GPIIb/IIIa itself. The function of integrins, such as GPIIb/IIIa, is regulated through multiple extracellular divalent cation binding sites. However, GPIIb/IIIa can be activated by inside-out signaling [26]. Therefore, as a result of a non-physiological stimulus, we could detect increased activation of GPIIb/IIIa, since we used the special activation-dependent monoclonal antibody. On the other hand, stimulation with the ionophore increased PS expression. PS expression in the extracellular membrane of platelets gives it procoagulant characteristics. This is because lipid transport in the membrane is calcium-dependent [27].

The present study shows that microparticles produced by the effect of NAcGlc expressed a decrease in P-selectin compared to microparticles generated without an agonist and with ICa. We do not know if the canonical platelet pathway, which induces granule secretion, is not involved. However, NAcGlc increases GPIIb/IIIa significantly more than microparticles produced with no agonist and thrombin. Furthermore, the translocation of PS to the membrane surface of microparticles generated by NAcGlc was greater than in microparticles produced with thrombin and ICa. These findings demonstrate that NAcGlc-generated MPs are distinct from ADP- and ICa-generated MPs, and that they exhibit high GPIIb/IIIa expression and likely have aggregation capacity. It is known that the addition of sugars such as NAcGlc modifies proteins. It has been shown that modifying proteins by sugars affects numerous cellular functions [28]. There are in vitro studies showing that platelet contact with NAcGlc polymers induces shape change, pseudopod extension, and complex formation with integrins through calcium signaling [29]. A scanning and confocal microscopy study showed that platelet contact with p-NAcGlc increased intracellular calcium and the expression of P-selectin, GPIIb/IIIa, and PS [20]. In another study using platelet-rich plasma, the addition of p-NAcGlc slightly increased PS expression and increased PMP production [30]. On the other hand, several studies prove that NAcGlc has an anti-inflammatory capacity in various conditions, such as osteoarthritis and respiratory viral infections [18]. It has even been tested in polymeric form in improving wound healing in conditions such as diabetes [31,32] and for its participation in the immune response [33]. The results of the present study suggest that NAcGlc probably interacts with glycan-binding proteins, i.e., glycoproteins expressed on the platelet membrane.

The generation of MPs by the different agonists presented different phenotypes. This can be attributed to the fact that platelets have numerous receptors on their surfaces that perform diverse functions, and the specific activation of each of these receptors generates varied effects. Thrombin is a potent agonist that activates the platelet receptors PAR1 and PAR4. Through the incision of these receptors, thrombin activates the G protein pathway in the membrane, which activates the PLCβ pathway and subsequently generates PIP3, leading to an increase in cytosolic Ca^+2^ and, consequently, granule secretion, resulting in P-selectin expression on MP. In the case of ADP, it is known that it activates the platelet through the P2Y1 and P2Y12 receptors, which are coupled to G proteins; these activate PLCβ and PIK3, respectively, for the generation of PIP3 and, subsequently, the induction of Ca^+2^ release. This pathway stimulates the expression and activation of GPIIb/IIIa [34]. ICa A23187 is a non-physiological agonist. It is known that platelet activation occurs through the activation of channels that allow for the passage of divalent cations into the cell, resulting in the mobilization of intracellular calcium, an increase in cytosolic calcium, and an increase in calcium stores. This leads to GPIIb/IIIa activation, granule secretion, and PS expression in MPs.

NAcGlc is a sugar whose activation pathway is unknown; the MP phenotype exhibited increased GPIIb/IIIa activation and PS expression. We suggest that one possible signaling pathway is through the P2Y1 and P2Y12 receptors. The latter receptor exhibits prolonged activity, resulting in increased GPIIb/IIIa expression. In addition, the CLEC2 receptor, a C-lectin-like receptor, may also be involved. It recognizes podoplanin, a sialoprotein that induces activation through protein tyrosine kinases, involving the SFK and SYK proteins, resulting in the activation of PI3K and PLCγ, and the release of Ca^+2^ into the cytoplasm. Still, it may be recognized by receptors such as GPVI and CLEC2.

As we already mentioned, the differences in signaling pathways involve G proteins, which form a very diverse family of subunits that couple according to the signals received, producing responses that differ in magnitude, duration, and structural changes in the receptors. Different platelet receptors are involved in the generation of different PM phenotypes. Although all of them lead to calcium mobilization, the activation pathways are different. Not only are there different platelet subpopulations [35], but there are also different PM phenotypes that depend on the inducing stimulus that generated them.

Platelets play a crucial role in controlling bleeding. However, excessive aggregation can contribute to clotting in vessels, such as coronary arteries, and lead to heart infarction. In this work, we demonstrate that platelet-derived microparticles induced by different agonists have distinct effects on cultured endothelial cells. In other words, not all platelet-derived microparticles are identical. In the present study, we induce in vitro microparticles from platelets. However, the phenotype of microparticles may differ in distinct pathologies, depending on the stimulus that causes them. For example, acute inflammation, such as that caused by myocardial infarction, can induce distinct types of microparticles compared to chronic inflammation, like chronic heart failure, due to a different cytokine milieu [6,23]. This observation could be extrapolated to other microparticles derived from distinct cells, such as monocytes, lymphocytes, or endothelial cells themselves.

### 3.3. Effect of Microparticles on the Expression of Endothelial Cell Adhesion Molecules

Although the increased expression of GPIIb/IIIa and PS on the microparticle membrane in response to some agonists could confer aggregating and procoagulant capacity to the microparticles, GPIIb/IIIa and PS are not involved in the expression of adhesion molecules in the endothelium directly. Our results show an increase in the expression of endothelial adhesion molecules when stimulated with MPs generated by different agonists, all from platelets; however, the response was not the same. We do not know the content of the MPs, but it is known that inside the platelets there are granules that may contain cytokines such as TNF-α, IL-1, and CD40L, among other cytokines, have a role as inducers of the expression of endothelial adhesion molecules [36]. During platelet activation, they are expelled to the outside through secretion through the microparticles.

Endothelial cells were activated when stimulated with all five groups of microparticles. The expression levels of E-selectin, ICAM-1, VCAM-1, and PECAM-1 increased significantly compared to baseline.

We found that endothelial cells increased the expression of E-selectin when stimulated with microparticles generated with thrombin, ICa, and NAcGlc compared to the basal. The expression of E-selectin with thrombin, ICa, and NAcGlc was increased similarly to stimulation with TNF-α. In contrast, E-selectin expression was very low with the stimulation of microparticles derived with no agonist and with ADP. E-selectin is a glycoprotein of the selectin family, which is expressed in the endothelium. This selectin is transcriptionally expressed from the stimulus by TNF, IL-1, LPS [37], and CD40L. It has been proven that the CD40L molecule expressed on platelets by thrombin activation induces E-selectin expression in umbilical cord vein endothelial cells [38].

E-selectin expression also increased significantly when endothelial cells were stimulated with NAcGlc-generated MPs. Macrophages stimulated in vitro with oligochitosan, a compound composed of between four and ten N-acetylglucosamine or glucosamine residues, induced the production of TNF-α and IL-1β [39].

ICAM-1 expression in cells increased significantly compared to control cells when stimulated with microparticles generated with no agonist, thrombin, and NAcGlc. Although ICAM-1 increased significantly compared to baseline, the expression level was low compared to the positive control. The ICAM-1 molecule is a surface glycoprotein that constitutively has a low expression, which increases in response to stimuli with TNF-α or IL-1β [40] for stronger adhesion of leukocytes and their transmigration through the endothelium [41]. In this work, the content of the MPs was not determined, but it is known that the expression of ICAM-1 in endothelial cells increases when treated with thrombin-activated platelets [42]. It has also been reported that ICAM-1 expression is regulated by the activity of microRNAs (miRNA) [43]. It has been described that platelets may contain various miRNAs [44] and that they are transferred to endothelial cells to regulate gene expression [45]. It has been reported that platelet exosomes, with miRNA 223 [46], 141 [47], and 223 [48], activated with thrombin suppress the expression of ICAM-1.

VCAM-1 expression was significantly increased in endothelial cells with all five microparticle groups compared to baseline. ICa-generated microparticles had a more significant impact on VCAM-1 expression. Although the expression of ICAM-1 in the endothelium by MPs is clear, we do not know the activation pathway, since it has been reported that Vascular adhesion molecule-1 (VCAM-1), like ICAM-1, is involved in the firm adhesion of leukocytes to the apical surface of endothelial cells through interaction with the leukocyte receptor [49]. VCAM-1 expression is activated by pro-inflammatory cytokines such as TNFα, IL-1, ROS, oxidized low-density lipoproteins, high glucose concentration, toll-like receptor agonists, and shear stress [50,51].

When we measured PECAM-1 expression, we found that only microparticles generated with no agonist stimulated an increase in its expression compared to basal and TNF-α stimulation. Unlike the effect with microparticles produced by ADP, PECAM-1 expression was significantly decreased compared to microparticles with no agonist and TNF-α.

Our results on PECAM-1 expression varied for each group of generated MPs. This molecule is involved in many molecular processes; we do not know the content of the MPs, since it is known that PECAM-1 expression can have an activating or cellular transmigration function.

The PECAM-1 molecule, in addition to its role as an adhesion molecule, is involved in inflammatory responses and signaling, with essential roles in vascular biology such as angiogenesis, platelet function and thrombosis, mechano-sensitivity of the endothelial cell response to fluid shear stress, and the regulation of multiple stages of leukocyte movement through venular walls. Furthermore, PECAM-1 activates signaling pathways and interacts with the cytoskeleton; the dimerization of the extracellular domain confers outside-in and inside-out signaling of the cell to various stimuli. It has been reported that IL-1β is among the cytokines that mediate PECAM-1 expression [52].

Previous studies report that the combination of TNF-α and INF-γ can reduce the expression of PECAM-1 in the junctions of endothelial cells [53]. On the other hand, it has been reported that, in the content of the MPs generated by ADP, there are abundant membrane and soluble proteins related to platelet degranulation and the electron transport chain, as well as a smaller number of proteins associated with the organization of the cytoskeleton, as well as other proteins that participate in the activation.

The MPs are generated by different agonists, which produce different MP phenotypes, which are unknown in terms of their content and composition. On the other hand, endothelial activation is carried out by the consecutive expression of the following molecules: initially selectins, followed by integrins, ICAM-1, and VCAM-1. The expression of VCAM-1 is a marker of endothelial activation. However, the ligands for P-selectin and GPIIb/IIIa molecules are PSGL-1 and vWF, respectively, and PS, which binds to the cell membrane. MPs generated by ICa presented a higher expression of them, such that all contributed to the stimulation of endothelial cells, showing a stronger expression of VCAM-1. In contrast, with MPs generated by TH, only the P-selectin pathway was sufficient for VCAM-1 expression. On the other hand, MPs generated by ADP only contained GPIIb/IIIa, although they did activate the endothelium, but this was to a lesser extent. For NAcGlc-generated MPs presenting GPIIb/IIIa and PS, E-selectin expression was highest, but not sufficient. Possibly, the ligand of GPIIb/IIIa, vWF, is released from the membrane and remains in solution. Therefore, VCAM-1 expression occurs to a lesser extent. It is worth noting that recognition between receptors and their ligands is regulated by the magnitude, duration, and structural changes in signaling pathways.

### 3.4. Potential Clinical Implications

Anti-aggregation therapy is crucial in patients at risk of developing a myocardial infarction and other procoagulant conditions [28]. Interestingly, NAcGlc induces microparticles with a high expression of GPIIb/IIIa on their surface. Still, endothelial activation is similar to that of ADP, as measured by VCAM-1 expression on the endothelial cells. However, NacGlc induces more expression of E-selectin than ADP, suggesting that the mechanism of induction of microparticles and their content is different. Competition experiments with platelets using different antiaggregants are necessary to evaluate the action mechanism of NacGlc over platelets more closely. A NacGlc polymer is used as a hemostatic, but, in its non-polymeric form, it has been used as an anti-inflammatory agent and for other purposes [54]. Further experiments are necessary to study the potential uses of NacGlc in other pathological conditions.

### 3.5. Study Limitations

In this work, we did not evaluate the microparticle content, so we did not measure the presence of pro-inflammatory cytokines, proteins, or lipids in the microparticle experiments. Additionally, new studies on the signaling pathways involving specific receptors, such as PAR and P2Y (thrombin and ADP, respectively), are required. We only isolated the MPs by differential centrifugation, without analyzing them by size. The characterization of the MPs’ size distribution is an interesting topic for further studies. Since in vitro studies have limitations, the use of HMEC-1 cells enables a homogenized response to different stimuli, as it preserves the phenotypic characteristics of an endothelium, including the expression of adhesion molecules. However, the use of primary cultures would provide a more realistic approach; the variation in response, according to the biological characteristics of each individual, would influence the results. With our findings, we observed different responses of the endothelium to MPs of the same origin generated by different agonists. However, to assess the generalization of the present findings into clinical scenarios, including acute myocardial infarction, further studies are needed.

## 4. Materials and Methods

### 4.1. Study Design

**Figure 6:**
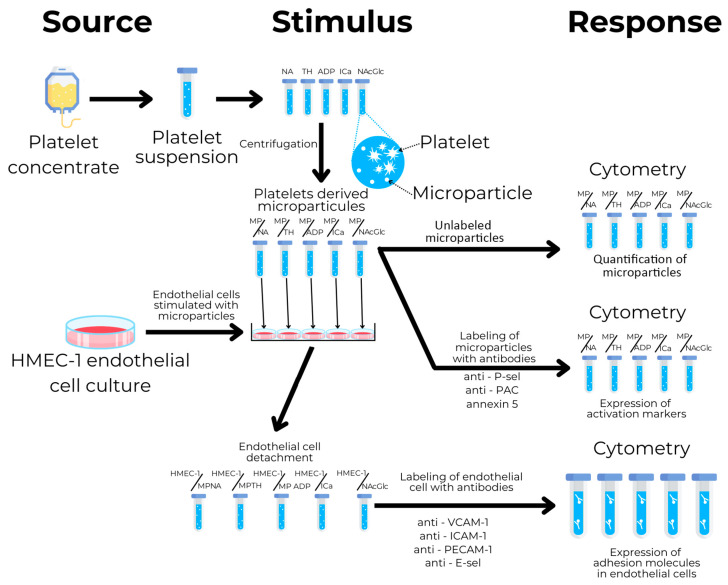
Diagram of the study design. Platelets were stimulated with thrombin (TH), ADP, calcium ionophore (ICa), N-acetylglucosamine (NAcGlc), and without activating (NA). Subsequently, the platelets were centrifuged to isolate the microparticles (MPs) produced by each stimulus: MP/NA, MP/TH, MP/ADP, MP/ICa, and MP/NAcGlc. The amounts of isolated microparticles were measured using cytometry (i.e., quantification). Also, the isolated microparticles were labeled with specific antibodies for P-selectin, GPIIb/IIIa, and annexin V, and were measured using cytometry. Endothelial cells of the HMEC-1 line were cultured in five groups and stimulated with the microparticles generated by the different agonists. The cells were detached and labeled with specific antibodies for E-selectin, ICAM-1, VCAM-1, and PECAM-1, and were measured using cytometry.

The study design is illustrated in Figure 6. The first step was platelet collection from donor platelet concentrate samples. Platelets were pooled under five conditions: NA (no agonist), TH (thrombin), ADP (adenosine diphosphate), ICa (calcium ionophore A 23187), and NAcGln (N-acetylglucosamine), all from Sigma-Aldrich (Millipore-Sigma. St. Louis, MO, USA), and were stimulated with the corresponding agonists. The microparticles were subsequently isolated using centrifugation, labeled with antibodies specific for P-selectin, GPIIb/IIIa, and annexin 5 for phosphatidylserine, and quantified using flow cytometry. Endothelial cells from the cell line HMEC-1 were then cultured and stimulated with microparticles generated by different agonists. Stimulated endothelial cells were labeled with antibodies specific for ICAM-1, VCAM-1, PECAM-1, and E-selectin, and were measured using flow cytometry.

### 4.2. Platelet Collection

Apheresis-derived platelet concentrates were obtained from 23 healthy volunteer donors, comprising 21 men and 2 women, with an age range of 18–65 years. The inclusion criteria follow the guidelines outlined in the protocol for blood donation at our institution. The most relevant criteria include the level of hematocrit according to the altitude of residence above sea level (m): between 0 and 1500 m, hematocrit in men > 40% and in women > 38%; and for residence above 1501 m, hematocrit in men > 44% and in women > 40%. Regarding the platelet count, it must be ≥150 × 10^9^/L. Participants were excluded if they had received five or fewer days of treatment with anticoagulant medication, including acetylsalicylic acid, Clopidogrel, Diflunisal, Phenylbutazone, Meloxicam, Nabumetone, Naproxen, Piroxicam, Sulindac, and Tenoxicam. Additionally, patients treated with anti-inflammatory drugs within the last 48 h were excluded, including Aceclofenac, Acetaminophen, Mefenamic acid, Diclofenac, Dexibuprofen, Flurbiprofen, Ibuprofen, Indomethacin, Ketoprofen, and Ketorolac. Participants gave their written informed consent. This study was approved by the Ethics Committee of the Ignacio Chávez National Institute of Cardiology (number 22-1343).

Platelets were processed within 60 min of collection. Apheresis-derived platelet concentrate was quantified using an automated cell counter (Cell-Dyn, Ruby Abbott Hematology), and three groups of platelets were formed to assess their functionality: unstimulated, thrombin-activated platelets, and ADP-activated platelets. Briefly, platelets were incubated for 10 min in citrate buffer (13 mM sodium citrate, 30 mM glucose, 120 mM sodium chloride, pH 6.5) with 1 U/mL of thrombin and 10 µM ADP. Platelets were centrifuged at 67× *g*/min (centrifuge 5415, Eppendorf, Westbury, NY, USA). P-selectin expression was measured using flow cytometry (FACSCalibur, Becton and Dickinson, San Jose, CA, USA) and analyzed with BD Cell Quest pro software version 5 (San Jose, CA, USA).

### 4.3. Microparticle Isolation

Platelets were adjusted to a concentration of 250 × 10^6^ platelets/mL in a sodium citrate solution. Five tubes were prepared with the same platelet concentration. Thrombin (1 U/mL), ADP (10 μM), calcium ionophore (5 μM), and N-acetylglucosamine (NAcGlc) (25 μM) were added to each platelet tube. A sample remained unstimulated and was incubated for 60 min at 37 °C. The thrombin agonist and ICa concentrations were taken from the literature [55]. To determine the optimal concentration of NAcGlc, we tested the following NAcGlc concentrations: 12.5 μM, 25 μM, 50 μM, and 100 μM. The maximum expression of P-selectin on the surface of platelets labeled with APC-coupled anti-CD62 antibody was reached at 25 μM of NAcGlc.

At the end of the 60 min stimulation period, we added an equal volume of buffer to dilute the agonists to half their original concentration. Microparticle isolation from each group of stimulated platelets was as follows: activated platelets were removed by centrifugation at 604× *g* for 5 min, followed by 1 min at 5433× *g*. Each supernatant was transferred to another 1.5 mL tube and centrifuged at 67× *g* for 15 min to pellet cell debris and remove the remaining platelets. To obtain the MPs, the supernatant was transferred to another 1.5 mL tube and centrifuged at 13,148× *g* for 60 min. All the supernatant was eliminated and the MP pellet was recovered and resuspended in 200 µL of PBS. The concentration of the obtained MP was normalized for characterization. Briefly, the isolated MPs were resuspended in 200 µL. Then, 5 µL were taken to quantify MPs by flow cytometry using a 1-µm standard fluorescent bead (Megamix, BioCytex, Marseille, France).

To illustrate the gating strategy, an example of the flow cytometry analysis of platelets is shown in the Supplementary Materials, Figure S2, panel A. The dot plot of platelets without stimulation shows a homogeneous distribution in the individual platelet sizes (FSC-A) versus their complexity (SSC-A). Microparticles were labeled with an anti-CD41-APC antibody and Annexin V (Annexin V-FITC, Trevigen, Helgerman Court, Gaithersburg, MD, USA) to measure phosphatidylserine and adjust the acquisition parameters. In the Supplementary Materials, Figure S2, panel B, an example of the flow cytometry analysis of MPs labeled with CD41-APC is shown. The expression of CD41-APC of individual MPs above the auto-fluorescence line (i.e., 10^1^) verifies that MPs are derived from platelets.

### 4.4. Microparticle Characterization

From each of the five isolated MP populations (microparticles from non-stimulated platelets, i.e., MP/NA; thrombin-generated microparticles, MP/throm; ADP-generated microparticles, MP/ADP; calcium ionophore-generated microparticles, MP/ICa; and N-acetylglucosamine-generated microparticles, MP/NAcGlc), the expression of P-selectin (anti-CD62P-PE R&D MN, USA), GPIIb/IIIa (PAC-1-FITC, BD Bioscience San Diego, CA, USA), and phosphatidylserine (Annexin V-FITC, TREVIGEN Helgerman Court, Gaithersburg, MD, USA) was measured. The samples were incubated for 30 min at room temperature, protected from light. PBS (150 µL) was added and measured using cytometry.

### 4.5. Microparticle-Stimulated Endothelial Cells

HMEC-1 (human microvascular endothelial cells) were cultured in 24-well plates (Corning, NY, USA) in MCDB 131 culture medium (SIGMA, St. Louis, MO, USA), supplemented with 10 mM glutamine, a mixture of antibiotics (penicillin, streptomycin, and amphotericin B) (GIBCO, Grand Island, NY, USA), 10% fetal bovine serum (Hyclone, Logan, UT, USA), and 20 µg/mL endothelial growth factor. The confluent endothelial cells were washed with HEPES solution (150 mM NaCl, 4.4 mM KCl, 10 mM HEPES, 12.2 mM glucose, pH 7.5), and fresh culture medium was added. Subsequently, the MP suspension was added to each well. The MP concentration for stimulation in HMEC-1 cells was 4 × 10^5^ MP/500 mL per agonist (MP/NA, MP/TH, MP/ADP, MP/ICa, MP/NAcGlc), and the stimulus with TNFα at a concentration of 10 ng/mL was used as a positive control. The stimulus lasted four hours (for E-selectin) and 18 h (for ICAM-1, VCAM-1, and PCAM-1) at 37 °C with 7% CO_2_. The culture medium was removed, and the cells were washed with HEPES solution and detached with a collagen-trypsin-EDTA mixture (Sigma, Ronkonkoma, NY, USA) in a 2:1 ratio, and centrifuged at 67× *g*; the supernatant was removed, and the cells were washed with PBS/Albumin 0.8%; the cells were pelleted by centrifugation at 67× *g*, and the supernatant was decanted. The cell pellet was resuspended in PBS/Albumin.

### 4.6. Analysis of Endothelial Activation Markers

To analyze the effect of each MP group on endothelial cells, the expression of E-selectin, ICAM-1, VCAM-1, and PECAM-1 was measured with FITC-coupled anti-ICAM-1, PE-coupled anti-VCAM-1, FITC-coupled anti-PECAM-1, and PE-coupled anti-E-selectin antibodies (all from R&D, Minneapolis, MN, USA). The endothelial cell suspension stimulated with each MP population was separated into 25 µL aliquots, bringing them to a total volume of 50 µL with PBS/albumin, and incubated for 30 min at room temperature, protected from light. The cells were washed with PBS/albumin, centrifuged, and measured using cytometry to ensure 5000 events per sample. In flow cytometry, the entire endothelial cell population (HMEC-1) was analyzed, as it is a cell line. No gates were created. Labeling was performed with a single fluorochrome per tube for each sample, with no multiple labels used. The analysis strategy was the same under all conditions. The Supplementary Materials present an example of the dot plot representation of the flow cytometry gating strategy for endothelial cells (Figure S3). Also, it includes representative flow cytometry histograms of the expression of endothelial adhesion molecules across all agonists (Figure S1).

### 4.7. Statistical Analysis

The adhesion molecule expression experiments included the platelets from 23 healthy donors. After the elimination of data points from individual determinations that gave an out-of-range value, or in which the number of endothelial cells counted using cytometry did not reach 5000 events per sample, the sample sizes were as follows: E-selectin (N = 21), ICAM-1 (N = 19), VCAM-1 (N = 17), and PECAM-1 (N = 23). The datasets of adhesion molecule expression were tested for normal distribution using the Shapiro–Wilks test (Supplementary Materials, Table S8). Since most variables did not have a normal distribution, the data is shown as the median with an interquartile range and individual points. To optimize visualization due to potential skew in the expression of adhesion molecules, their plots are presented on a logarithmic scale (Figure 2, Figure 3, Figure 4 and Figure 5). The median expression values of molecules (P-Sel, GPIIb/IIIa, and PS) in the membrane of the MPs in response to the stimulus of the different agonists and the expression of adhesion molecules (E-selectin, ICAM-1, VCAM-1, and PECAM-1) to the stimulus with MPs generated by platelets and different agonists were compared using Kruskal–Wallis and Mann–Whitney U tests. To quantify the increase in expression of different molecules and adhesion molecules, the median fold change was calculated for the response to each agonist compared to the response with no agonist (for MP membrane molecules) and compared to the response at baseline conditions (for adhesion molecule expression). A *p*-value < 0.05 was considered significant. After multiple pair comparisons, the adjusted *p*-value was calculated using the Bonferroni method, i.e., by dividing the *p*-value of 0.05 by the number of multiple pair comparisons. This approach yielded adjusted *p*-values for statistical significance with 10 pair comparisons (*p* < 0.005) or with 21 pair comparisons (*p* < 0.0024). SPSS version 21.0 was used for statistical analysis.

## 5. Conclusions

Platelet-derived microparticles exhibit a distinct composition of GPIIb/IIIa and PS in the microparticle membrane, depending on the agonist that stimulates the platelets. The elevated expression of GPIIb/IIIa and PS in the microparticle membrane suggests that the microparticles have a pro-aggregate and procoagulant capacity. Moreover, these microparticles upregulate, in a different manner, the expression of the adhesion molecules E-selectin, VCAM-1, ICAM-1, and PECAM-1 in endothelial cells. These results demonstrate that platelet-derived microparticles induce stimulus-dependent responses in endothelial cells in response to various physiological and non-physiological stimuli, and confirm that not all microparticles, even if they are of the same origin—in this case, platelets—induce the same response, and that the role of microparticles on different diseases can vary depending on the stimulus that originated them. Platelet microparticles may play a role in modulating inflammation and other endothelial functions.

## Figures and Tables

**Table 1:** Summary of the median fold-change in the membrane surface molecules expressed on the platelet microparticles in response to stimuli with different agonists. The arrows indicate statistically significant change (adjusted *p*-value < 0.005) compared to the expression obtained with microparticles without an agonist.

Stimulus	P-Selectin	GPIIb/IIIa	PS
Thrombin	1.1 ↑	2.2	0.8
ADP	1.0	5.2 ↑	1.3
ICa	1.3 ↑	7.0 ↑	3.3 ↑
NAcGlc	0.8	11.4 ↑	3.0 ↑

PS = phosphatidylserine; ADP = adenosin dyphospate; ICa = calcium ionophore; NAcGlc = N-acetylglucosamine.

**Table 2:** Summary of the median fold-change in the adhesion molecules expressed on the endothelial cells in response to stimuli with microparticles (MPs) derived from platelets stimulated by different agonists. The arrows indicate statistically significant changes (adjusted *p*-value < 0.003) compared to the expression obtained without stimulation (baseline).

Stimulus	E-Selectin	ICAM-1	VCAM-1	PECAM
MPs derived from no agonist	1.4 ↑	3.0 ↑	15.8 ↑	1.7
MPs derived from thrombin	4.2 ↑	2.5 ↑	24.4 ↑	1.2
MPs derived from ADP	1.9 ↑	2.3 ↑	20.3 ↑	1.0
MPs derived from ICa	5.0 ↑	2.2 ↑	35.6 ↑	1.5 ↑
MPs derived from NAcGlc	5.2 ↑	2.4 ↑	18.8 ↑	1.2

ADP = adenosin dyphospate; ICa = calcium ionophore; NAcGlc = N-acetylglucosamine.

## Data Availability

The raw data supporting this article’s conclusions will be made available upon request to the corresponding author, provided that the pertinent legal requirements are met.

## Supplementary Materials

The following supporting information can be downloaded at: https://www.mdpi.com/article/10.3390/ijms26146567/s1.

## Abbreviations

The following abbreviations are used in this manuscript:

NA	No agonist
TH	Thrombin
ADP	Adenosine diphosphate
ICa	Calcium ionophore
NAcGlc	N-acetylglucosamine
MP	Microparticle
P-Sel	P-selectin
PS	Phosphatidylserine
GPIIb/IIIa	Glycoprotein IIb/IIIa
E-Sel	E-selectin
ICAM-1	Intercellular adhesion molecule 1
VCAM-1	Vascular cell adhesion molecule 1
PECAM-1	Platelet endothelial cell adhesion molecule 1
HMEC-1	Human microvascular endothelial cells 1
TNF-α	Tumor necrosis factor alpha
MCDB131	Molecular, cellular, and developmental biology 131 medium
HEPES	4-(2-hydroxyethyl)piperazine-1-ethanesulfonic acid
EDTA	Ethylenediaminetetraacetic acid
PBS	Phosphate-buffered saline
