# Supplementary material for: Effect of Platelet-Derived Microparticles on the Expression of Adhesion Molecules in Endothelial Cells
Source: Int J Mol Sci. 2025 Jul 8;26(14):6567. doi: 10.3390/ijms26146567 (PMC12294387; doi:10.3390/ijms26146567)
Supplement: Supplementary file 1 [file ijms-26-06567-s001.zip › ijms-3687729-supplementary.pdf]

Supplementary Material

Table S1. Comparisons of the median value of platelet-derived microparticle expression of P-selectin after platelets were stimulated by different agonists: Thrombin, ADP, Ica, and NAcGln (N = 6).

Agonist	Median (P25 – P75)	p-values			
		No agonist vs	Thrombin vs	ADP vs	ICa vs
No Agonist	14820 (14452 – 15187)				
Thrombin	16560 (15643 – 19443)	0.004*			
ADP	15023 (12285 – 19219)	0.747	0.336		
ICa	19272 (16181 – 21619)	0.004*	0.335	0.109	
NAcGln	11891 (11891 – 13840)	0.010	0.004*	0.149	0.004*

* Significant difference (adjusted p < 0.005; p = 0.05 / 10 comparisons), Mann-Whitney U test.

Table S2. Comparisons of the median value of platelet-derived microparticle expression of GPIIb/IIIa after platelets were stimulated by different agonists: Thrombin, ADP, Ica, and NAcGln (N = 6).

Agonist	Median (P25 – P75)	p-values			
		No agonist vs	Thrombin vs	ADP vs	ICa vs
No Agonist	6181 (1800 – 6181)				
Thrombin	13370 (10566 – 15770)	0.076			
ADP	32044 (30794 – 33987)	0.004*	0.004*		
ICa	433010 (41236 – 43905)	0.004*	0.004*	0.004*	
NAcGln	70207 (66133 – 84227)	0.004*	0.004*	0.037	0.054

* Significant difference (adjusted p < 0.005; p = 0.05 / 10 comparisons), Mann-Whitney U test.

Table S3. Comparisons of the median value of platelet-derived microparticle expression of phosphatidylserine (PS) after platelets were stimulated by different agonists: Thrombin, ADP, Ica, and NAcGln (N = 6).

Agonist	Median (P25 – P75)	p-values			
		No agonist vs	Thrombin vs	ADP vs	ICa vs
No Agonist	13872 (11184 – 14274)				
Thrombin	10517 (5382 – 11857)	0.109			
ADP	17934 (17934 – 19819)	0.100	0.044		
ICa	45562 (43910 – 45562)	0.004*	0.004*	0.006	
NAcGln	41925 (27464 – 56430)	0.004*	0.004*	0.010	0.518

* Significant difference (adjusted p < 0.005; p = 0.05 / 10 comparisons), Mann-Whitney U test.

Table S4. P-values from comparisons of the median value of E-selectin expression measured from the membrane of endothelial cells after exposure to microparticles generated by platelets stimulated by different agonists: Thrombin, ADP, Ica, and NAcGln (N = 21).

		p-values					
				Exposure to platelet-derived microparticles			
**Agonist**	**Median (P25 – P75)**	Baseline vs	TNF-α vs	No agonist vs	Thrombin vs	ADP vs	ICa vs
Baseline	2706 (2706 – 2706)						
TNF-α	17137 (17137 – 17137)	< 0.001*					
No agonist	3737 (3737 – 3737)	< 0.001*	< 0.001*				
Thrombin	11260 (8951 – 11260)	< 0.001*	< 0.001*	< 0.001*			
ADP	5264 (3271 – 5264)	0.001*	< 0.001*	0.023	< 0.001*		
ICa	13411 (9670 – 13411)	< 0.001*	0.001*	< 0.001*	0.082	< 0.001*	
NAcGln	14148 (7040 – 20772)	< 0.001*	0.090	< 0.001*	0.132	< 0.001*	0.317

* Significant difference (adjusted p-value < 0.0024; p = 0.05 / 21 comparisons), Mann-Whitney U test.

Table S5. P-values from comparisons of the median value of ICAM-1 expression measured from the membrane of endothelial cells after exposure to microparticles generated by platelets stimulated by different agonists: Thrombin, ADP, Ica, and NAcGln (N = 19).

		p-values					
				Exposure to platelet-derived microparticles			
**Agonist**	**Median (P25 – P75)**	Baseline vs	TNF-α vs	No agonist vs	Thrombin vs	ADP vs	ICa vs
Baseline	10889 (9647 – 10889)						
TNF-α	286501 (279201 – 286501)	< 0.001*					
No agonist	32477 (23320 – 42964)	< 0.001*	< 0.001*				
Thrombin	27157 (17437 – 37085)	< 0.001*	< 0.001*	0.286			
ADP	25308 (24087 – 30216)	< 0.001*	< 0.001*	0.025	0.569		
ICa	24205 (20062 – 28697)	< 0.001*	< 0.001*	0.019	0.549	0.492	
NAcGln	26609 (20039 – 41119)	0.001*	< 0.001*	0.328	0.782	0.439	0.193

* Significant difference (adjusted p-value < 0.0024; p = 0.05 / 21 comparisons), Mann-Whitney U test.

Table S6. P-values from comparisons of the median value of VCAM-1 expression measured from the membrane of endothelial cells after exposure to microparticles generated by platelets stimulated by different agonists: Thrombin, ADP, Ica, and NAcGln (N = 17).

		**p-values**					
				Exposure to platelet-derived microparticles			
**Agonist**	**Median (P25 – P75)**	Baseline vs	TNF-α vs	No agonist vs	Thrombin vs	ADP vs	ICa vs
Baseline	369 (350 – 369)						
TNF-α	32687 (32687 – 32687)	< 0.001*					
No agonist	5850 (4825 – 6274)	< 0.001*	< 0.001*				
Thrombin	8998 (4963 – 12823)	< 0.001*	< 0.001*	0.065			
ADP	7493 (5057 – 9235)	< 0.001*	< 0.001*	0.152	0.326		
ICa	13154 (11916 – 17067)	< 0.001*	< 0.001*	< 0.001*	0.020	0.002*	
NAcGln	6953 (6125 – 8986)	< 0.001*	< 0.001*	0.022	0.185	0.796	< 0.001*

* Significant difference (adjusted p-value < 0.0024; p = 0.05 / 21 comparisons), Mann-Whitney U test.

Table S7. P-values from comparisons of the median value of PECAM-1 expression measured from the membrane of endothelial cells after exposure to microparticles generated by platelets stimulated by different agonists: Thrombin, ADP, Ica, and NAcGln (N = 23).

		**p-values**					
				Exposure to platelet-derived microparticles			
**Agonist**	**Median (P25 – P75)**	Baseline vs	TNF-α vs	No agonist vs	Thrombin vs	ADP vs	ICa vs
Baseline	3282 (3282 – 3808)						
TNF-α	3645 (3081 – 3645)	0.135					
No agonist	5689 (2603 – 6017)	0.024	0.088				
Thrombin	3968 (2324 – 3968)	0.127	0.096	0.136			
ADP	3175 (2575 – 3175)	0.037	0.004	0.004	0.007		
ICa	4928 (3811 – 5867)	< 0.001*	0.004	0.834	0.05	< 0.001*	
NAcGln	4014 (3410 – 4666)	0.029	0.031	0.092	0.124	0.001*	0.061

* Significant difference (adjusted p-value < 0.0024; p = 0.05 / 21 comparisons), Mann-Whitney U test.

Table S8. P-values from the Shapiro-Wilk tests for Normal distribution applied to the expression levels of endothelial adhesion molecules after exposure of microparticles generated by platelets stimulated by different agonists.

Stimulus	ESEL (N = 21)	ICAM (N = 19)	VCAM (N = 17)	PECAM (N = 23)
Baseline	< 0.001*	< 0.001*	0.021*	0.010*
TNF-α	0.001*	< 0.001*	< 0.001*	0.001*
No agonist	< 0.001*	0.562	0.004*	0.014*
Thrombin	0.040*	0.442	0.028*	0.010*
ADP	0.106	0.101	0.606	0.016*
ICa	0.018*	0.179	0.276	0.017*
NacGln	0.091	0.052	0.464	0.056

* The data has no Normal distribution ($p < 0.05$)

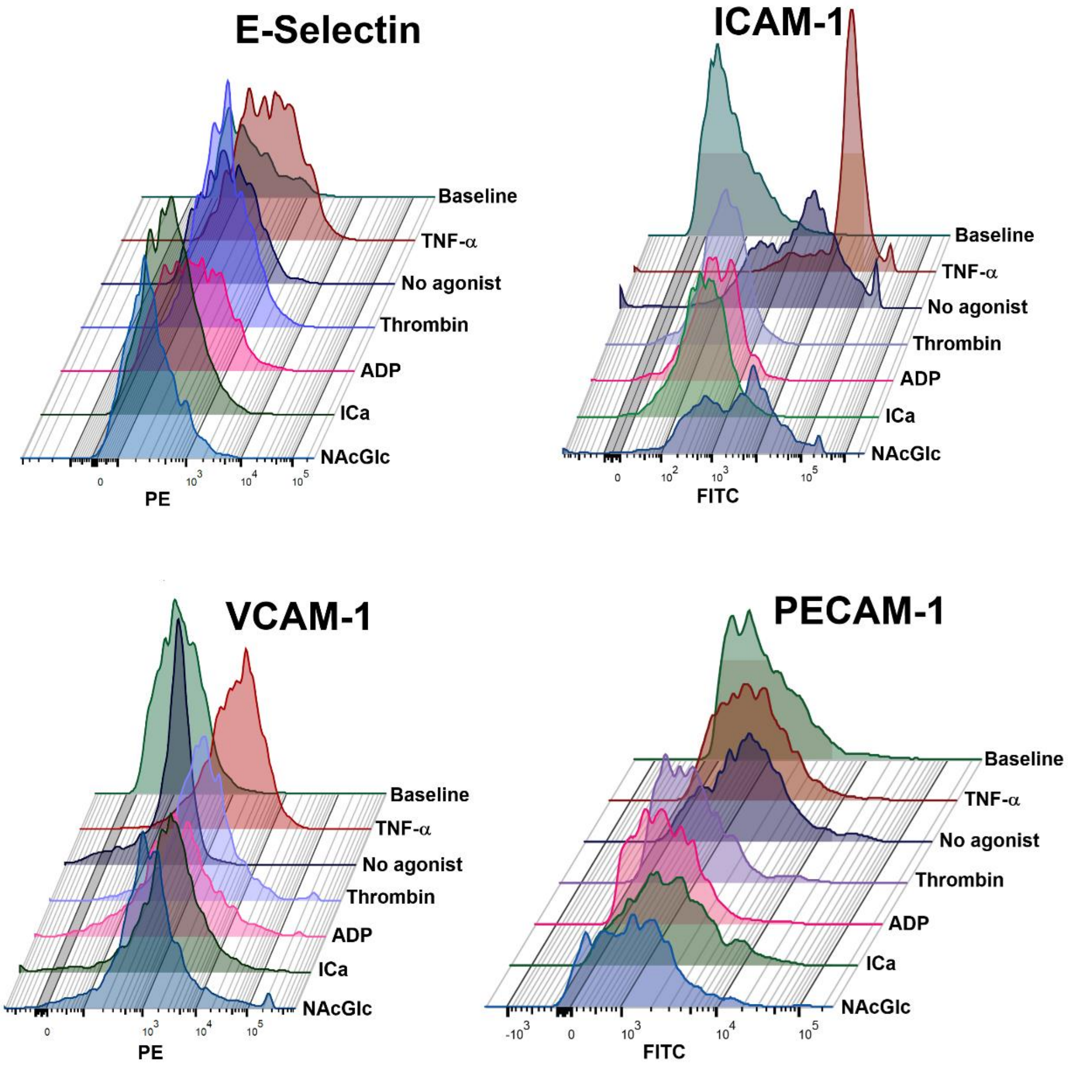

Figure S1. Representative flow cytometry histograms of the expression of endothelial adhesion molecules (E-Selectin, ICAM-1, VCAM-1, and PECAM-1) without stimulation (baseline), with stimulation (TNF-α), and upon exposure to microparticles derived from platelets activated with different agonists (no agonist, thrombin, ADP, ICa, and NAcGlc).

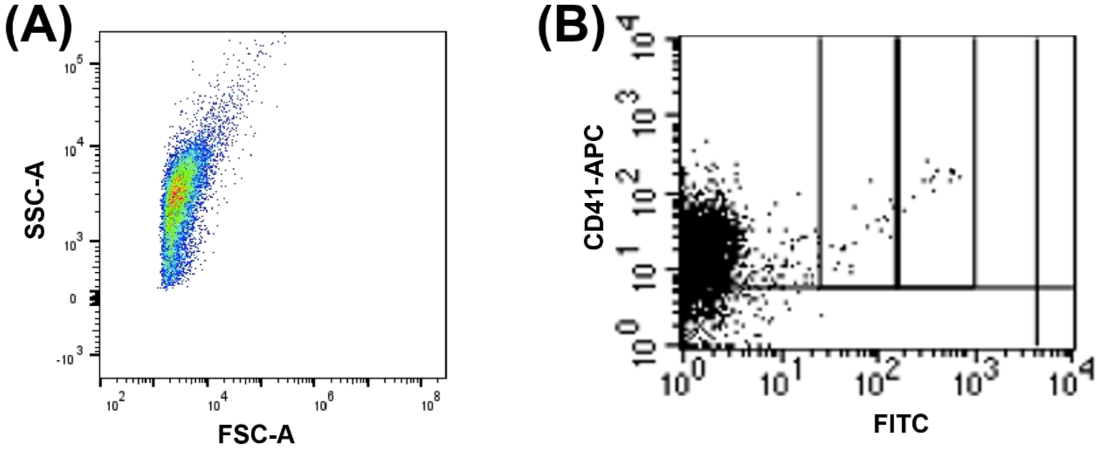

Figure S2. Representation of the dot plot analysis by flow cytometry of platelets from a healthy donor (A) and microparticles derived from platelets subsequently labeled with CD41-APC (B).

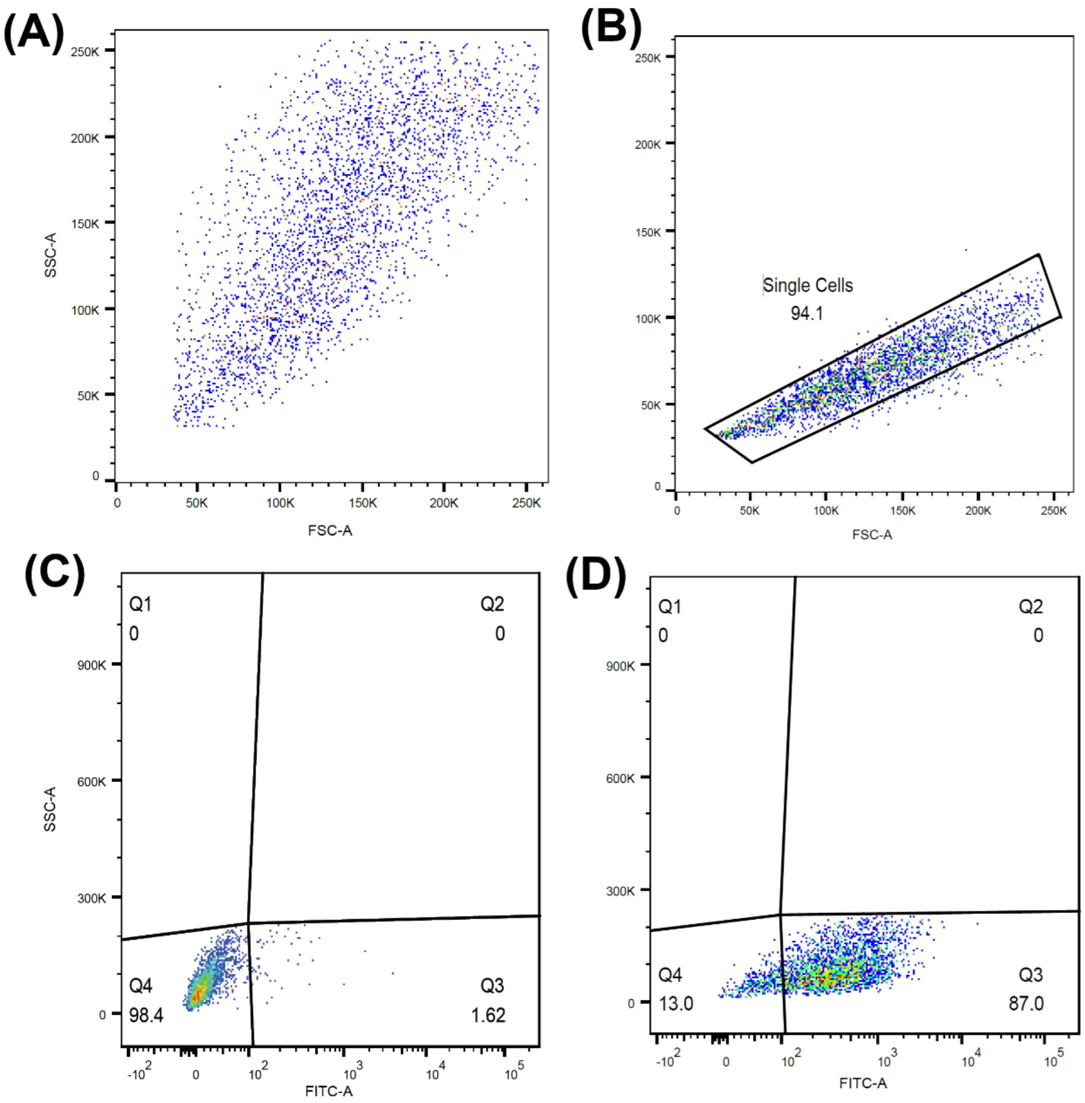

Figure S3. Dot plot representation of the flow cytometry gating strategy of endothelial cells: Acquisition of HMEC-1 cells (A), Single cells of HMEC-1 (B), unstimulated cells of HMEC-1 (C), labeled cells showing the expression of VCAM-1 in cells stimulated with MP derived from platelets exposed to ICa (D).
